# Baseline hematology and serum biochemistry results for Indian leopards (*Panthera pardus fusca*)

**DOI:** 10.14202/vetworld.2017.818-824

**Published:** 2017-07-24

**Authors:** Arun Attur Shanmugam, Sanath Krishna Muliya, Ajay Deshmukh, Sujay Suresh, Anukul Nath, Pa Kalaignan, Manjunath Venkataravanappa, Lyju Jose

**Affiliations:** 1 Department of Biotechnology, Jain University, Bengaluru, Karnataka, India; 2 Wildlife SOS, Bengaluru, Karnataka, India; 3 Bannerghatta Biological Park, Bannerghatta, Bengaluru, Karnataka, India; 4 Manikdoh Leopard Rescue Center, Wildlife SOS, Khamgaon, Maharashtra, India; 5 Department of Ecology & Environmental Science, E. P. Odum School of Environmental Sciences, Silchar, Assam, India; 6 Wild Animal Disease Diagnostic Lab, Bannerghatta Biological Park, Bannerghatta, Bengaluru, Karnataka, India

**Keywords:** hematology, Indian leopard, *Panthera pardus fusca*, serum biochemistry

## Abstract

**Aim:**

The aim of the study was to establish the baseline hematology and serum biochemistry values for Indian leopards (*Panthera pardus fusca*), and to assess the possible variations in these parameters based on age and gender.

**Materials and Methods:**

Hemato-biochemical test reports from a total of 83 healthy leopards, carried out as part of routine health evaluation in Bannerghatta Biological Park and Manikdoh Leopard Rescue Center, were used to establish baseline hematology and serum biochemistry parameters for the subspecies. The hematological parameters considered for the analysis included hemoglobin (Hb), packed cell volume, total erythrocyte count (TEC), total leukocyte count (TLC), mean corpuscular volume (MCV), mean corpuscular Hb (MCH), and MCH concentration. The serum biochemistry parameters considered included total protein (TP), albumin, globulin, aspartate aminotransferase, alanine aminotransferase (ALT), blood urea nitrogen, creatinine, triglycerides, calcium, and phosphorus.

**Results:**

Even though few differences were observed in hematologic and biochemistry values between male and female Indian leopards, the differences were statistically not significant. Effects of age, however, were evident in relation to many hematologic and biochemical parameters. Sub-adults had significantly greater values for Hb, TEC, and TLC compared to adults and geriatric group, whereas they had significantly lower MCV and MCH compared to adults and geriatric group. Among, serum biochemistry parameters the sub-adult age group was observed to have significantly lower values for TP and ALT than adult and geriatric leopards.

**Conclusion:**

The study provides a comprehensive analysis of hematologic and biochemical parameters for Indian leopards. Baselines established here will permit better captive management of the subspecies, serve as a guide to assess the health and physiological status of the free ranging leopards, and may contribute valuable information for making effective management decisions during translocation or rehabilitation process.

## Introduction

Leopards are one of the widely distributed wild felids in the world, with nine subspecies found in varied habitats across their extent range [1]. The species is currently categorized as “vulnerable” on the IUCN Red List, with some subspecies identified as “endangered” or “critically endangered” [2]. India hosts substantial numbers of the subspecies *Panthera pardus fusca*, the Indian leopard which survives in protected areas and multiple use forests all across India, except for the arid deserts and regions above the timberline in the Himalayas [3]. The subspecies is even known to persist close to human populations by feeding on livestock and domestic dogs [4]. Ensuing this, incidences of human-leopard conflicts have also seen a sharp rise in recent past in India, leading to casualties on both sides. This has, in turn, led to increased veterinary interventions in the species. Further, a large population of Indian leopards exit in captivity maintained in almost all the Indian zoos [5].

Given the difficulty in detecting signs of disease and distress in wild animals, comprehensive health assessments, including hematology and serum biochemistry studies, have been crucial to assess the effects of many health-related problems [6]. Such information has also been proven useful to assess the subclinical effects of pathogens [7] and physiological [8,9], ecological [10], or nutritional [7] status, among other issues.

To interpret a laboratory report to be normal or abnormal, the values expected to be obtained from healthy animals (reference intervals) must be known first [11]. However, unlike other large felids [12-15], a reliable baseline hematological and serum biochemistry references, that can be used to assess the health status of Indian leopards is still lacking. The reference intervals available are usually based on limited population testing and do not account for variation within the subspecies or within subpopulations defined by age, gender, and other factors [16,17]. Further, these parameters can also be influenced by several additional extrinsic factors such as the presence of pathological condition, phlebotomy techniques used, and chemical immobilization before sampling [18]. Thus, this study was undertaken to establish the baseline reference interval of 7 hematology and 11 serum biochemistry variables from Indian leopards and to assess the influence of age and gender on these variables, while keeping the extrinsic factors constant.

## Materials and Methods

### Ethical approval

Not applicable: None of the animals were sampled solely for the purpose of this study. The study was carried out by compiling and analyzing available hemato-biochemical test reports, carried out as part of routine health evaluations in both the study sites.

### Study area and animals

Hemato-biochemical test reports from a total of 83 healthy leopards, carried out as part of routine health evaluation in Bannerghatta Biological Park, Karnataka (BBP, n=41) and Manikdoh Leopard Rescue Center, Maharashtra (MLRC, n=42) between January 2014 and January 2016 were used to establish baseline hematology and serum biochemistry parameters for the subspecies. The leopards from BBP included both captive born leopards from zoo section and rescued free ranging leopards from rescue center, whereas leopards from MLRC were mainly rescued free ranging leopards. While the reports from animals which appeared to be healthy and clinically stable during sampling were considered, reports from the ones with visible injuries and known history of illness were excluded from the analysis.

### Blood collection and processing

Since it is crucial that samples are obtained under consistent conditions to arrive at baseline value, the restraint and phlebotomy techniques were also considered. Based on available information, uniform protocols were followed in both the institutions. All the leopards considered for this study were physically restrained by securing them in squeeze cages, and phlebotomy was done from lateral coccygeal vein, without any chemical immobilization. Hematological parameters were evaluated using commercially available automated analyzers (BBP: HumaCount 30TS, HUMAN Gesellschaft für Biochemica und Diagnostica mbH, Wiesbaden, Germany; MLRC: BC-1800, Vector Biotek Pvt. Ltd. Gujarat, India), whereas the serum biochemistry assays were performed on semi-automated clinical chemistry analyzers (BBP: ERBA Chem Pro, Transasia House, Mumbai, India; MLRC: VChem+, Vector Biotek Pvt. Ltd. Gujarat, India) using commercially available biochemical kits, calibrated with control reagents before sample analysis. Further, all the samples were confirmed to have undergone laboratory analyses within 2-3 h of collection.

### Hemato-biochemical parameters

The hematological parameters considered for the analysis included hemoglobin (Hb in g/dl), packed cell volume (in %), total erythrocyte count (TEC in 10^6^/µl), total leukocyte count (TLC in 10^3^/µl), mean corpuscular volume (MCV in fl), mean corpuscular Hb (MCH in pg), and MCH concentration (in g/dL). The serum biochemistry parameters considered included total protein (TP in g/dl), albumin (in g/dl), globulin (in g/dl), aspartate aminotransferase (AST in IU/L), alanine aminotransferase (ALT in IU/L), blood urea nitrogen (in mg/dl), creatinine (in mg/dl), triglycerides (in mg/dl), calcium (in mg/dl), and phosphorus (in mg/dl).

### Statistical analysis

For the purpose of statistical analysis, leopards were grouped according to their gender (male, n=34 and female, n=49) and further into three age groups: Sub-adults (1-3 years old; n=19), adults (3-10 years old, n=38) and geriatric animals (10 years and above, n=26), based on available zoo/rescue center records. The mean and standard deviation for each hemato-biochemical values obtained were calculated as per the standard procedure [19]. If any value was less than or equal to the first quartile −3 times the interquartile range or greater than the third quartile +3 times the interquartile range, they were regarded as extreme outliers and were removed before further analyses and then the middle 95% of test results were considered as the reference interval. Data were further tested for normality using the Shapiro–Wilk test for normality using R version 3.2.5. The dataset which followed normal distribution was tested using independent t-test to compare the mean of the hematological parameters, whereas the parameters which did not meet the criteria of normality were tested using Mann–Whitney U-tests (two factors) to evaluate significant differences. The variation between different age classes irrespective of gender was tested using one-way ANOVA followed by Tukey test for the data following the normal distribution pattern and Kruskal–Wallis test for the data which did not meet the criteria. Significance level was p<0.05 for all tests.

## Results

The results and descriptive statistics for hemato-biochemistry parameters analyzed in this study are listed in Tables-1-4. Even though few differences were observed in hematologic and biochemistry values between male and female Indian leopards, the differences were statistically not significant.

**Table-1:** Overall hematology and serum biochemistry results for Indian leopards (*Panthera pardus fusca*) from BBP and MLRC.

Parameters	n	Minimum	Maximum	Mean±SD	SEM	95% CI of the mean	

						LCI	UCI
TLC (10^3^/µl)	83	8.0	36.9	16.2±6.1	0.7	14.9	17.5
TEC (10^6^/µl)	83	5.4	12.7	8.7±2.0	0.2	8.3	9.1
Hb (g/dl)	83	10.5	20.2	14.7±2.5	0.3	14.2	15.3
PCV (%)	83	32.8	65.4	48.5±6.8	0.8	47.0	50.0
MCV (fl)	83	45.9	93.9	58.6±11.3	1.2	56.2	61.1
MCH (pg)	83	13.6	28.0	18.1±3.0	0.3	17.4	18.7
MCHC (%)	83	21.7	36.0	30.7±2.8	0.3	30.1	31.3
TP (g/dl)	83	4.3	11.1	7.0±1.3	0.1	6.7	7.3
Albumin (g/dl)	83	1.6	7.0	3.9±1.1	0.1	3.7	4.1
Globulin (g/dl)	83	0.3	6.6	3.0±1.2	0.1	2.8	3.3
AST (IU/L)	41	9.9	90.8	41.3±19.2	3.0	35.5	47.2
ALT (IU/L)	83	2.0	146.7	44.0±28.2	3.1	37.9	50.0
CRT (mg/dl)	83	0.4	4.2	1.6±0.7	0.1	1.4	1.7
BUN (mg/dl)	83	3.1	56.3	27.6±11.4	1.3	25.1	30.0
Glucose (mg/dl)	41	14.1	159.0	58.6±26.9	4.2	50.4	66.9
Triglyceride (mg/dl)	41	4.0	59.9	29.5±12.5	2.0	25.6	33.3
Ca (mg/dl)	83	1.5	27.0	9.9±3.2	0.3	9.2	10.6
P (mg/dl)	83	2.2	12.4	5.5±1.8	0.2	5.1	5.9

TLC=Total leukocyte count, TEC=Total erythrocyte count, Hb=Hemoglobin, PCV=Packed cell volume, MCV=Mean corpuscular volume, MCH=Mean corpuscular hemoglobin, MCHC=Mean corpuscular hemoglobin concentration, TP=Total protein, AST=Aspartate aminotransferase, ALT=Alanine aminotransferase, BUN=Blood urea nitrogen, Ca=Serum calcium, P=Serum phosphorus, CI=Confidence interval, LCI=Lower confidence interval, UCI=Upper confidence interval, BBP=Bannerghatta Biological Park, MLRC=Manikdoh Leopard Rescue Center, SEM=Standard error of mean, SD=Standard deviation

**Table-2:** Comparison of hematology and serum biochemistry results for male and female Indian leopards (*Panthera pardus fusca*) from BBP and MLRC.

Parameters	Male							Female							p value
	
	n	Min	Max	Mean±SD	SEM	95% CI of the mean		n	Min	Max	Mean±SD	SEM	95% CI of the mean		
	
						LCI	UCI						LCI	UCI	
TLC (10^3^/µl)	34	8.7	36.9	17.2±6.5	1.1	15.0	19.4	49	8.0	28.2	15.5±5.9	0.8	13.9	17.0	0.37
TEC (10^6^/µl)	34	5.8	12.7	8.9±2.2	0.4	8.2	9.7	49	5.4	11.8	8.6±1.8	0.3	8.0	9.1	0.99
Hb (g/dl)	34	10.5	20.2	15.0±2.7	0.5	14.1	15.9	49	11.0	19.9	14.6±2.3	0.3	14.0	15.1	0.57
PCV (%)	34	32.8	65.4	49.2±7.2	1.2	46.8	51.6	49	34.0	60.7	48.0±6.6	0.9	46.2	49.7	0.83
MCV (fl)	34	47.0	88.1	57.5±11.1	1.9	53.8	61.2	49	45.9	93.9	59.4±11.6	1.7	56.0	62.7	0.45
MCH (pg)	34	14.2	28.0	18.3±3.3	0.6	17.2	19.4	49	13.6	25.0	17.9±2.8	0.4	17.1	18.6	0.59
MCHC (%)	34	24.4	35.1	31.1±2.3	0.4	30.3	31.9	49	21.7	36.0	30.4±3.0	0.4	29.6	31.1	0.46
TP (g/dl)	34	4.6	10.4	6.8±1.2	0.2	6.4	7.2	49	4.3	11.1	7.1±1.4	0.2	6.7	7.4	0.31
Albumin (g/dl)	34	1.9	7.0	4.0±1.1	0.2	3.6	4.4	49	1.6	6.6	3.9±1.1	0.2	3.5	4.2	0.46
Globulin (g/dl)	34	0.3	5.6	2.9±1.2	0.2	2.4	3.3	49	0.3	6.6	3.2±1.3	0.2	2.8	3.5	0.66
AST (IU/L)	22	10.6	90.8	44.7±21.0	4.5	35.9	53.4	19	9.9	77.5	37.5±16.8	3.9	29.8	45.1	0.24
ALT (IU/L)	34	2.0	120.2	37.0±22.9	3.9	29.3	44.7	49	3.7	146.7	48.8±30.6	4.4	40.1	57.4	0.91
Creatinine (mg/dl)	34	0.5	3.0	1.5±0.6	0.1	1.3	1.7	49	0.4	4.2	1.6±0.7	0.1	1.4	1.7	0.32
BUN (mg/dl)	34	3.9	56.3	27.5±13.6	2.3	22.9	32.1	49	3.1	49.9	27.6±9.7	1.4	24.8	30.3	0.63
Glucose (mg/dl)	22	14.1	159.0	59.4±30.7	6.6	46.6	72.2	19	14.1	95.1	57.7±22.4	5.1	47.7	67.6	0.81
Triglycerides (mg/dl)	22	4.7	59.9	31.1±12.0	2.6	26.1	36.1	19	4.0	52.9	27.6±13.2	3.0	21.7	33.4	0.39
Ca (mg/dl)	34	3.5	14.7	9.6±2.7	0.5	8.7	10.5	49	1.5	27.0	10.1±3.5	0.5	9.1	11.0	0.67
P (mg/dl)	34	2.4	12.4	5.6±2.3	0.4	4.8	6.3	49	2.2	8.9	5.5±1.5	0.2	5.1	5.8	0.70

TLC=Total leukocyte count, TEC=Total erythrocyte count, Hb=Hemoglobin, PCV=Packed cell volume, MCV=Mean corpuscular volume, MCH=Mean corpuscular hemoglobin, MCHC=Mean corpuscular hemoglobin concentration, TP=Total protein, AST=Aspartate aminotransferase, ALT=Alanine aminotransferase, BUN=Blood urea nitrogen, Ca=Serum calcium, P=Serum phosphorus, CI=Confidence interval, LCI=Lower confidence interval, UCI=Upper confidence interval, BBP=Bannerghatta Biological Park, MLRC=Manikdoh Leopard Rescue Center, SEM=Standard error of mean, SD=Standard deviation

**Table-3:** Comparison of hematology results for sub-adult, adult and geriatric Indian leopards (*Panthera pardus fusca*) from BBP and MLRC.

Parameters	n	Min	Max	Mean	SEM	SD	95% CI of the mean		p value

							LCI	UCI	
Sub-adults									
TLC (10^3^/µl)	19	10.3	26.4	19.1	0.9	4.1	17.2	20.9	0.027
TEC (10^6^/µl)	19	6.8	12.7	10.1	0.4	1.7	9.4	10.8	0.001
Hb (g/dl)	19	10.5	20.2	16.0	0.7	2.9	14.7	17.2	0.05
PCV (%)	19	32.8	65.4	49.9	1.9	8.4	46.1	53.6	0.537
MCV (fl)	19	47.0	54.2	50.8	0.5	2.1	49.8	51.7	0.002
MCH (pg)	19	14.2	18.4	16.3	0.2	1.1	15.8	16.8	0.009
MCHC (%)	19	29.7	33.6	31.9	0.1	1.2	33.7	34.0	0.112
Adults									
TLC (10^3^/µl)	38	8.7	27.9	15.8	1.1	6.8	13.6	17.9	-a
TEC (10^6^/µl)	38	5.4	11.8	8.4	0.3	1.9	7.8	9.0	-
Hb (g/dl)	38	11.0	20.2	14.4	0.4	2.1	13.7	15.1	-
PCV (%)	38	34.0	60.9	48.5	1.0	6.4	46.4	50.5	-
MCV (fl)	38	46.2	88.6	60.8	2.1	12.8	56.7	64.8	-
MCH (pg)	38	13.6	23.9	18.8	0.6	3.4	17.8	19.9	-
MCHC (%)	38	25.6	34.2	30.4	0.6	3.4	29.4	31.5	-
Geriatric									
TLC (10^3^/µl)	26	8.7	27.9	14.7	1.1	5.8	12.5	16.9	-a
TEC (10^6^/µl)	26	5.4	11.8	8.1	0.4	1.9	7.4	8.9	-
Hb (g/dl)	26	11.0	20.2	14.3	0.5	2.5	13.4	15.3	-
PCV (%)	26	34.0	60.9	47.6	1.2	6.3	45.1	50.0	-
MCV (fl)	26	46.2	88.55	61.3	2.1	10.7	57.1	65.4	-
MCH (pg)	26	13.6	23.88	18.2	0.5	2.8	17.1	19.3	-
MCHC (%)	26	25.6	34.2	30.3	0.5	2.4	29.3	31.2	-

^a^ p values for sub-adult, adult and geriatric group comparison are listed in the top (sub-adult) half of the table. TLC=Total leukocyte count, TEC=Total erythrocyte count, Hb=Hemoglobin, PCV=Packed cell volume, MCV=Mean corpuscular volume, MCH=Mean corpuscular hemoglobin, MCHC=Mean corpuscular hemoglobin concentration, CI=Confidence interval, LCI=Lower confidence interval, UCI=Upper confidence interval, BBP=Bannerghatta Biological Park, MLRC=Manikdoh Leopard Rescue Center, SEM=Standard error of mean, SD=Standard deviation

**Table-4:** Comparison of serum biochemistry results for sub-adult, adult and geriatric Indian leopards (*Panthera pardus fusca*) from BBP and MLRC.

Parameter	n	Minimum	Maximum	Mean	SEM	SD	95% CI of the mean		p value

							LCI	UCI	
Sub adult									
TP (g/dl)	19	4.3	11.1	6.5	0.3	1.4	5.9	7.2	0.01
Albumin (g/dl)	19	1.9	5.0	4.0	0.2	0.8	3.6	4.3	0.91
Globulin (g/dl)	19	0.3	6.6	2.7	0.3	1.4	2.0	3.3	0.168
AST (IU/L)	19	9.9	77.7	39.6	4.5	19.8	30.7	48.5	0.561
ALT (IU/L)	19	2.0	50.2	25.8	3.3	14.5	19.2	32.3	0.001
Creatinine (mg/dl)	19	0.4	4.2	1.7	0.2	1.0	1.2	2.1	0.213
BUN (mg/dl)	19	7.5	56.1	25.7	2.7	11.9	20.4	31.1	0.645
Glucose (mg/dl)	19	14.1	159.0	66.1	7.3	31.8	51.8	80.3	0.249
Triglycerides (mg/dl)	19	4.0	52.9	27.4	3.0	13.0	21.5	33.2	0.62
Ca (mg/dl)	19	3.5	27.0	9.3	1.1	5.0	7.1	11.6	0.193
P(mg/dl)	19	2.6	12.4	6.2	0.5	2.1	5.2	7.2	0.181
Adult									
TP (g/dl)	38	4.9	10.1	7.0	0.2	1.4	6.6	7.4	-^a^
Albumin (g/dl)	38	1.8	6.7	3.9	0.2	1.2	3.6	4.3	-
Globulin (g/dl)	38	0.3	5.2	3.0	0.2	1.2	2.6	3.4	-
AST (IU/L)	16	13.7	49.0	45.3	5.2	20.7	35.1	55.4	-
ALT (IU/L)	38	21.3	147.0	46.8	4.4	26.8	38.3	55.3	-
Creatinine (mg/dl)	38	0.5	2.5	1.7	0.1	0.6	1.5	1.8	-
BUN (mg/dl)	38	3.1	56.3	27.5	1.8	11.4	23.9	31.1	-
Glucose (mg/dl)	16	24.1	66.2	53.5	5.6	22.5	42.4	64.5	-
Triglycerides (mg/dl)	16	22.9	43.9	31.1	3.4	13.6	24.4	37.8	-
Ca (mg/dl)	38	7.3	14.2	10.0	0.4	2.5	9.2	10.8	-
P(mg/dl)	38	2.22	12.4	5.4	0.3	1.6	4.9	5.9	-
Geriatric									
TP (g/dl)	26	4.9	10.08	7.3	0.2	1.2	6.9	7.8	-^a^
Albumin (g/dl)	26	1.8	6.7	3.8	0.2	1.0	3.5	4.2	-
Glo (g/dl)	26	0.3	5.23	3.4	0.2	1.1	2.9	3.8	-
AST (IU/L)	6	13.7	49	36.5	5.5	13.6	25.7	47.4	-
ALT (IU/L)	26	21.3	146.7	53.1	6.3	32.1	40.8	65.5	-
Creatinine (mg/dl)	26	0.5	2.54	1.4	0.1	0.5	1.2	1.6	-
BUN (mg/dl)	26	3.1	56.3	29.0	2.2	11.3	24.6	33.3	-
Glucose (mg/dl)	6	24.1	66.2	48.9	6.0	14.8	37.1	60.8	-
Triglycerides (mg/dl)	6	22.9	43.9	31.7	3.0	7.3	25.8	37.5	-
Ca (mg/dl)	26	7.3	14.21	10.1	0.4	2.2	9.3	11.0	-
P(mg/dl)	26	2.2	12.4	5.2	0.4	1.9	4.5	5.9	-

a p values for sub-adult, adult and geriatric group comparison are listed in the top (sub-adult) half of the table. TP=Total protein, AST=Aspartate aminotransferase, ALT=Alanine aminotransferase, BUN=Blood urea nitrogen, Ca=Serum calcium, P=Serum phosphorus, CI=Confidence interval, LCI=Lower confidence interval, UCI=Upper confidence interval, BBP=Bannerghatta Biological Park, MLRC=Manikdoh Leopard Rescue Center, SEM=Standard error of mean, SD=Standard deviation

Effects of age, however, were evident in relation to many hematologic and biochemical parameters. Sub-adults had significantly greater values (mean±standard deviation) for Hb (16.0±2.9 g/dL), TEC (10.1±1.7 × 10^6^/µl), and TLC (19.1±4.1 × 10^6^/µl) compared to adults (Hb: 14.4±2.1 g/dL; TEC: 8.4±1.9 × 10^6^/µl and TLC: 15.8±6.8 × 10^3^/µl) and geriatric group (Hb: 14.3±2.5 g/dL; TEC: 8.1±1.9 × 10^6^/µl and TLC: 14.7±5.8 × 10^3^/µl), whereas they had significantly lower MCV (50.8±2.1 fl) and MCH (16.3±1.1 pg) compared to adults (MCV: 60.8±12.8 fl and MCH: 18.8±3.4 pg) and geriatric group (MCV: 61.3±10.7 fl and MCH: 18.2±2.8 pg). Among, serum biochemistry parameters the sub-adult age group were observed to have significantly lower values for TP (6.5±1.4 g/dl) and ALT (25.8±14.5 IU/L) than adults (TP: 7.0±1.4 g/dl and ALT: 46.8±26.8 IU/L) and geriatric leopards (TP: 7.3±1.4 g/dl and 53.1±32.1 IU/L) age groups.

## Discussion

Flagging hematology and biochemistry values obtained from an animal as either normal or abnormal is the first interpretive step in interpreting hemato-biochemical reports. However, this is not as simple as it may seem in wild animals as reference intervals are usually based on limited population testing and do not account for variation within subpopulations defined by age, sex, subspecies, or extrinsic factors such as instrumentation and phlebotomy techniques. Further, adequate numbers of normal animals must be sampled to arrive at intervals that are valid for healthy animals from the defined population. Thus, unlike published studies [16], sampling large numbers of animals to make the results most reflective of the healthy population is highly desirable.

A reference interval is typically defined as values encompassing the median 95% of a tested population of apparently healthy animals [11]. Inherent in this definition is that 2.5% of the healthy population will have values outside either side of the median 95%, suggesting they are abnormal [11]. Thus, the nonparametric method used to arrive at reference intervals in this study, wherein the test values were rank ordered, and any outliers were removed and then the middle 95% of test results define the reference interval are less biased, compared to other methods [9,11,20].

In felids, the number, size and Hb concentration of circulating erythrocytes is known to increase gradually after 1 month of age and, at 3-4 months reaches values similar to those of adults depending on the amount of iron in the diet [21]. However, this study showed significantly greater TEC and Hb counts in sub-adults compared to adult and geriatric animals. This might be subsequent to transient polycythemia from splenic contraction [22], a momentary response to epinephrine due to stress, anger, and fear as observed in sub-adults, most of them with a history of being and the RBC counts are known to revert to normal in a short period [22].

Higher TLC observed in sub-adults may also be indicative of low-level stressor factors inherent with the animals newly introduced to captive environment. The significantly lesser MCV and MCH values observed in sub-adults, in comparison to geriatric and adult age groups leopards can be attributed to the age and dietary differences that permit older age classes to produce relatively larger erythrocytes than younger age classes; similar to the patterns observed in other carnivores [20,23,24].

Among serum biochemistry parameters, sub-adults had significantly lower TP compared to adults and geriatric leopards. The increased serum concentrations of TPs in the older age groups could be explained as an age-related phenomenon, having been observed in other carnivores [25,26] and also probably due, in part, to the increase in gammaglobulins induced by either vaccinations or increased contact with environmental microorganisms [27]. Further, the TP levels for immature and growing animals tend to be normally low, consistent with the expected higher physiological demand for proteins during their growth phase [28].

ALT and AST are used as general indicators of liver function, with ALT being more liver-specific [29]. In this study, older age classes were observed to have higher ALT activity compared to young animals and in particular, sub-adults had significantly lower ALT levels than geriatric animals. This observation in older age classes can be attributed to physiological variations related with age, hormone action, reproductive phases as well as exposure of liver to a wide array of toxins, infectious, agents, drug metabolites and endotoxins over time [30].

## Conclusion

This study provides a comprehensive analysis of hematologic and biochemical parameters for Indian leopards, including the possible variations in these parameters based on age and sex. To the best of authors’ knowledge, there are no similar published reference intervals available for the subspecies and thus the baselines provided here will permit better captive management of the subspecies, serve as a guide to assess the health and physiological status of the free ranging leopards, and may contribute valuable information for making effective management decisions during translocation or rehabilitation process.

## Authors’ Contributions

AAS and SKM conceived and designed the study. AAS, SKM, AD, SS and PK were involved in collection and compilation of data from BBP and MLRC. MV and LJ performed the hematology serum biochemistry analysis. AN carried out all the statistical analysis required for the manuscript. AAS, SKM, AD, SS, PK, MV, AN, and LJ contributed to the manuscript writing and the reviewing of the literature. All authors read and approved the final manuscript.
